# Isolation, Characterization, and Antioxidant Activity Evaluation of a Fucoidan from an Enzymatic Digest of the Edible Seaweed, *Hizikia fusiforme*

**DOI:** 10.3390/antiox9050363

**Published:** 2020-04-27

**Authors:** Lei Wang, Thilina U. Jayawardena, Hye-Won Yang, Hyo Geun Lee, Min-Cheol Kang, K. K. Asanka Sanjeewa, Jae Young Oh, You-Jin Jeon

**Affiliations:** 1 Department of Marine Life Sciences, Jeju National University, Jeju, Jeju Self-Governing Province 63243, Korea; comeonleiwang@163.com (L.W.); tuduwaka@gmail.com (T.U.J.); koty221@naver.com (H.-W.Y.); hond0502@hanmail.net (H.G.L.); networksun@naver.com (M.-C.K.); asanka.sanjeewa001@gmail.com (K.K.A.S.); 2 Marine Science Institute, Jeju National University, Jeju, Jeju Self-Governing Province 63333, Korea; 3 Research Group of Process Engineering, Korea Food Research Institute, Wanju, Jeollabuk-do 55365, Korea

**Keywords:** *Hizikia fusiforme*, fucoidan, oxidative stress, vero cells, zebrafish

## Abstract

The previous study suggested that the sulfated polysaccharides from *Hizikia fusiforme* (HFPS) possess strong antioxidant activity. The purpose of this study is to isolate fucoidan from HFPS and to investigate its antioxidant activity. A fucoidan (HFPS-F4) with a molecular weight of 102.67 kDa was isolated from HFPS. HFPS-F4 contains 99.01% of fucoidan (71.79 ± 0.56% of carbohydrate and 27.22 ± 0.05% of sulfate content). The fucoidan increased the viability of H_2_O_2_-treated Vero cells by 5.41, 11.17, and 16.32% at the concentration of 12.5, 25, and 50 μg/mL, respectively. Further results demonstrated that this effect act diminishing apoptosis by scavenging intracellular reactive oxygen species (ROS) via increasing the expression of the endogenous antioxidant enzymes, which was induced by elevating total nuclear factor (erythroid-derived 2)-like 2 (Nrf2) levels. In addition, the in vivo test results displayed that the pretreatment of fucoidan improved the survival rates and decreased heart-beating rate, ROS, cell death, and lipid peroxidation in H_2_O_2_-stimulated zebrafish. Taken together, these results demonstrated that fucoidan isolated from HFPS has strong in vitro and in vivo antioxidant activities and it could be utilized in pharmaceutical, nutraceutical, and cosmeceutical industries.

## 1. Introduction

Oxidative stress is an imbalance condition between the intracellular reactive oxygen species (ROS) production and scavenging in the body [1]. Generally, ROS are naturally produced during the aerobic metabolism and scavenged by the antioxidant defense system. However, the balance between ROS generation and scavenging could be broken down by the environmental stresses such as chemical, fine dust particles, and ultraviolet irradiation [2,3,4,5]. An excess of ROS could damage cellular organelles and biomacromolecules, including proteins, lipids, and nucleic acids [6]. The accumulation of intracellular damage leads to cell dysfunction and further induces various diseases, including Alzheimer disease, abnormal aging, cancer, diabetes, inflammation, and liver injury [7]. To discover the compounds with strong ROS scavenging activity but non-toxicity, thus, may be a realistic strategy for prevention or treatment of the diseases caused by oxidative stress. Owing to the advantages of natural compounds with high bioactivities and non-toxicity, isolating bioactive compounds from terrestrial and marine organisms have attracted attention in scientific research [1].

Seaweeds are an excellent bio-resource, which contain various bioactive compounds, including polyphenols, polysaccharides, proteins, sterols, and pigments [8]. In specific, seaweeds contain high amounts of polysaccharides, which possess numerous bioactivities, including anti-cancer, anti-coagulant, antioxidant, anti-inflammation, anti-diabetes, and anti-hypertension activities [9,10]. Fucoidans, the sulfated polysaccharides isolated form brown seaweeds, possess strong antioxidant activity [11]. Numerous of studies suggested that fucoidans possess strong in vitro and in vivo antioxidant activities [11,12,13].

*Hizikia fusiforme* (*H. fusiforme*), a popular edible brown seaweed, has been used as food and medicine in Asian countries such as China, Korea, and Japan for thousands of years. *H. fusiforme* contains various bioactive compounds including polyphenols, sterols, and polysaccharides [13]. In particular, *H. fusiforme* contains high amount of polysaccharides, which possess numerous bioactivities, such as anti-cancer, anti-viral, antioxidant, immune-modulating, and UV protective effects [14,15]. The previous study suggested that the sulfated polysaccharides isolated from *H. fusiforme* (HFPS) possesses strong antioxidant activity in in vitro and in vivo models [16]. However, the polysaccharide of HFPS has not been further purified and characterized. The objectives of this study are as follow: to isolate a fucoidan from HFPS; character the structural characteristics of the fucoidan; investigate the in vitro and in vivo antioxidant activities of the fucoidan.

## 2. Materials and Methods

### 2.1. Reagents and Chemicals

Penicillin-streptomycin, trypsin-EDTA, Roswell Park Memorial Institute-1640 (RPMI-1640) medium, and fetal bovine serum (FBS) were purchased from Gibco-BRL (Grand Island, NY, USA). Potassium bromide (KBr), Celluclast (≥700 units/g), 2,2-azobis (2-amidinopropane) hydrochloride, deuterium oxide (D_2_O), 3-(4,5-dimethylthiazol-2-yl)-2,5-diphenyltetrazolium bromide (MTT), 1-diphenyl-2-picrylhydrazyl (DPPH), trifluoroacetic acid, glucose, arabinose, fucose, galactose, glucose, and rhamonose were purchased from Sigma-Ardrich Co. (St. Louis, MO, USA). Other chemicals and reagents used in the present study were analytical grade.

### 2.2. Extraction and Isolation

The crude sulfated polysaccharides of *H. fusiforme* were prepared following the previous studies [16]. In brief, the lyophilized seaweed powder was hydrolyzed by Celluclast (5% enzyme, pH 4.5, and 50 ℃) for 24 h. The enzyme was inactivated (100 ℃, 10 min) and the pH was adjusted to neutral. Then, we got the Celluclast extract of *H. fusiforme* and referred as HF. HF was precipitated by ethanol, then the crude polysaccharides were obtained and referred as HFPS.

HFPS was loaded to a DEAE-cellulose column and eluted by NaCl containing sodium acetate (50 mM, pH 5.0). The elution was carried out with a gradient of NaCl (0~2 M, 30 mL/h). Each 10 mL eluent was collected, and the polysaccharide content of each fraction was measured using phenol–H_2_SO_4_ assay. The polysaccharide fractions were pooled, dialyzed, and freeze-dried.

### 2.3. Free Radical Scavenging Activity

DPPH, hydroxyl, and alkyl radical scavenging activities of each polysaccharides isolated from HFPS were evaluated using an Electron Spin Resonance (ESR) spectrometer (JES-FA machine; JEOL, Tokyo, Japan) using the method described by Heo et al. [17].

### 2.4. Chemical Composition Analysis

The total phenolic and carbohydrate contents of the target fraction (HFPS-F4) were measured according to the protocol of the Association of Official Analytical Chemists (AOAC) [18]. The sulfate content of HFPS-F4 was measured by the method described by Wang et al. [16]. The neutral sugar component of HFPS-F4 was determined by high-performance anion-exchange chromatography with pulsed amperometric detection (HPAE-PAD) base on the procedure described in the previous studies [19].

### 2.5. Chemical Characterization of HFPS-F4

#### 2.5.1. Fourier-Transform Infrared (FT-IR) Analysis

The IR spectra of HFPS-F4 and the commercial fucoidan (Sigma-Ardrich Co.) were recorded using a FT-IR spectrometer (Nicolet™ 6700 FT-IR spectrometer; Madison, WI, USA). HFPS-F4 and commercial fucoidan were separately homogenized with KBr, and then the mixture were pressed into pellets for FT-IR measurement in the frequency range of 500~2000 cm^−1^.

#### 2.5.2. Molecular Weight Analysis

The molecular weight (Mw) of HFPS-F4 was analyzed by high-performance gel permeation chromatography (HPGPC) with two types of size-exclusion chromatography columns in series, namely TSKgel 2500PW_xl_ (7.8 × 300 mm, Tosoh Co., Ltd., Tokyo, Japan) and TSKgel GMPW_xl_ (7.8 × 300 mm, Tosoh Co., Ltd., Tokyo, Japan), on a Waters HPLC system (Waters, USA) equipped with a Waters 2414 differential refractive index detector [20]. The HFPS-F4 solution (3 mg/mL, 200 μL) was filtered through a Nylon filter (0.45 μm). Filtered HFPS-F4 solution (20 μL) was injected into the column and eluted with NaNO_3_ (0.1 M, 1 mL/min; 60 min, 40 °C). The Mw of HFPS-F4 was calculated by the calibration curve obtained using various standard dextran.

#### 2.5.3. H-NMR Spectral Analysis

HFPS-F4 (30 mg) was dissolved in D2O (1 mL) in a NMR tube and analyzed in JEOL JNM-ECX400 spectrometer (Japan) and the chemical shifts were expressed in parts per million (ppm) [21].

### 2.6. Cell Culture

Vero cells (monkey kidney fibroblasts) were maintained in FBS (10%), streptomycin (100 µg/mL), and penicillin (100 unit/mL) supported RPMI-1640 medium. Cells were sub-cultured every 3 days and seeded at a density of 1 × 105 cells/mL for experiments.

### 2.7. Effect of Fucoidan on H_2_O_2_-Stimulated Vero Cells

#### 2.7.1. Measurement of Cell Viability and Intracellular ROS Level

To measure the effect of fucoidan on H_2_O_2_-induced cytotoxicity, Vero cells were pretreated with 12.5, 25, and 50 µg/mL of fucoidan for 1 h and incubated with H_2_O_2_ for 24 h. The viability of H_2_O_2_-induced Vero cells was measured by MTT assay, according to Wang et al. [22]. For measuring the intracellular ROS level, Vero cells were pretreated with fucoidan for 1 h, then, H_2_O_2_ (1 mM) was added to the cells. After 1 h, DCFH-DA (500 µg/mL, stock) was added to the cells. The fluorescence emission of DCF-DA was detected using a fluorescence microplate reader (BioTek, Synergy, HT, USA).

#### 2.7.2. Measurement of Apoptotic Body’s Formation

To assess the apoptosis stimulated by H_2_O_2_, Vero cells were treated with fucoidan and stimulated with H_2_O_2_ (1 mM) for 6 h. The H_2_O_2_-stimulated Vero cells were stained by Hoechst 33342. H2O2-treated Vero cells were stained by Hoechst 33342 based on the protocol described by Wijesinghe et al. [23]. Apoptosis levels were measured using Image J software.

#### 2.7.3. Western Blot Assay

The effect of fucoidan on catalase (CAT), superoxide dismutase-1 (SOD-1), and nuclear factor (erythroid-derived 2)-like 2 (Nrf2) levels were assessed by Western blot analysis performed as described previously [24]. Vero cells were seeded and treated with fucoidan for 1 h. The fucoidan-treated cells were stimulated with H_2_O_2_ for 6 h, and then the cells were harvested to investigating CAT, SOD-1, and Nrf2 levels. The amount of CAT, SOD-1, and Nrf2 were compared with β-actin.

### 2.8. Effect of Fucoidan on H_2_O_2_-Stimulated Zebrafish

Approximately 7~9 h post-fertilization (hpf), the embryos (15 embryos/group) were treated with fucoidan (12.5, 25, and 50 μg/mL). After 1 h, H_2_O_2_ (5 mM) was added to the embryos and then incubated until 24 hpf. The survival rates of H_2_O_2_-treated zebrafish were measured at three days post-fertilization (dpf) by counting the number of live zebrafish. The surviving zebrafish were used for further experiments.

At 2 dpf, the heart-beating rate of zebrafish was measured under the microscope based on the protocol described by Kim et al. [25]. The ROS, lipid peroxidation, and cell death of zebrafish were measured using DCFH-DA, DPPP, and acridine orange staining, respectively [26,27]. The relative fluorescence intensities of zebrafish were determined using Image J software.

### 2.9. Statistical Analysis

The experiments were conducted in triplicate and the data were expressed as the mean ± standard error (SE). One-way ANOVA was used to compare the mean values of each treatment in SPSS 12.0. Significant differences between the means were identified by the Tukey test.

## 3. Results and Discussion

### 3.1. Free Radical Scavenging Activities of the Polysaccharides Isolated from HFPS

**Figure 1:**
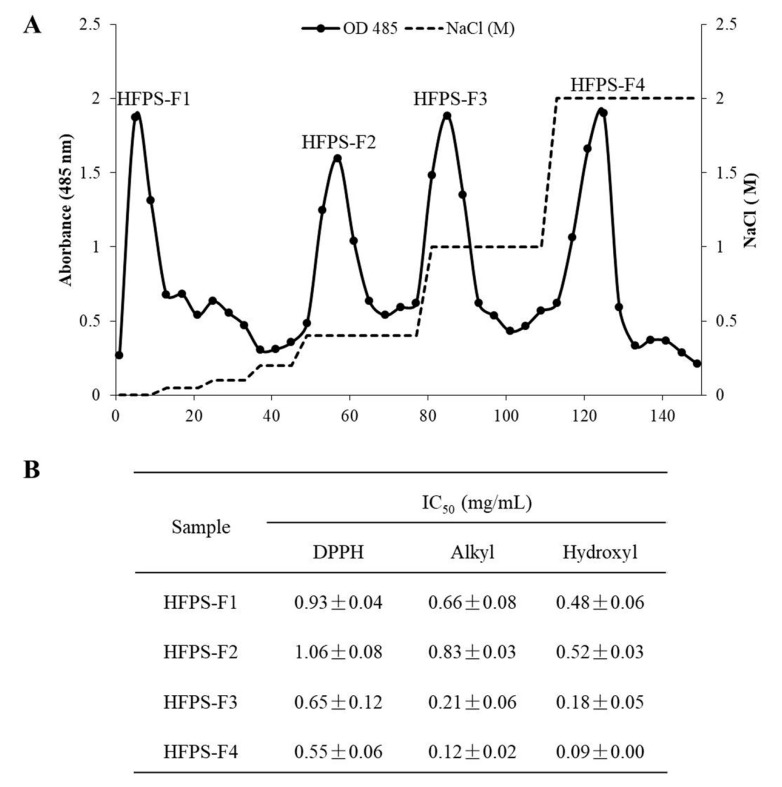
Isolation of polysaccharides from the crude sulfated polysaccharides of *H. fusiforme* (HFPS) and evaluation of their antioxidant activities. (**A**) The DEAE-cellulose chromatogram of the HFPS; (**B**) the free radical scavenging activities of polysaccharides isolated from HFPS. The experiments were conducted in triplicate, and the data are expressed as the mean ± standard error (SE).

The crude sulfated polysaccharides of *H. fusiforme* (HFPS) was obtained by Celluclast-assisted extraction and ethanol precipitation. HFPS was applied to a DEAE-cellulose column and eluted with NaCl. Four polysaccharides (HFPS-F1, HFPS-F2, HFPS-F3, and HFPS-F4) were separated according to the absorbance at 485 nm after phenol–H_2_SO_4_ assay, which measured the polysaccharide content of each fraction (Figure 1A). The antioxidant activities of these polysaccharides were investigated by evaluation of their free radical scavenging activities. As the results shown, these polysaccharides possess strong free radical scavenging activity and shown stronger effect on hydroxyl radical compared with another radicals (Figure 1B). In addition, HFPS-F4 possesses strongest free radical scavenging activity than other samples. It scavenged DPPH, alkyl, and hydroxyl radicals at the IC_50_ values of 0.55 ± 0.06, 0.12 ± 0.02, and 0.09 ± 0.00 mg/mL, respectively. According to these results, HFPS-F4 was selected as the target sample for further studies to analysis its chemical characteristics and to evaluate its antioxidant activity in vitro and in vivo models.

### 3.2. Chemical Composition and Structural Characteristics of HFPS-F4

In the present study, the phenolic, carbohydrate, and sulfate content of HFPS-F4 were determined as 0.00 ± 0.12, 71.79 ± 0.56, and 27.22 ± 0.05%, respectively (Table 1). The previous study indicated that the crude polysaccharides (HFPS) contain 1.56 ± 0.08% of phenolic content, 55.05 ± 0.09% of carbohydrate content, and 7.78 ± 0.23% of sulfate content, respectively [16]. These results demonstrated that HFPS-F4 contains almost non-phenolic content and high amount of carbohydrate and sulfate content, which suggested that the phenolic content was removed, while the carbohydrate and sulfate content were concentrated during separation by DEAE-cellulose column.

**Figure 2:**
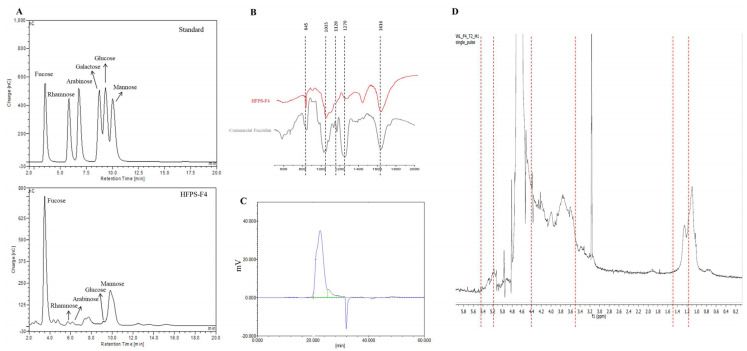
Chemical characterization of HFPS-F4. (**A**) High-performance anion-exchange chromatography (HPAE-PAD) spectrum of standard monosaccharide and HFPS-F4; (**B**) FT-IR spectra of HFPS-F4; (**C**) gel permeation chromatogram of HFPS-F4; (**D**) ^1^H NMR spectra of HFPS-F4.

In this study, the Mw of HFPS-F4 was determined via HPGPC (Figure 2C). The results indicated that the average Mw of HFPS-F4 is 102.67 kDa (Table 1). In addition, the monosaccharide composition of HFPS-F4 had been investigated (Figure 2A). The result indicated that the carbohydrate in HFPS-F4 is composed by 79.20% of fucose, 2.09% of rhamnose, 0.19% of glucose, 18.13% of mannose, and 0.38% of arabinose (Table 1). These results demonstrated that HFPS-F4 contains a high amount of carbohydrate (71.79 ± 0.56%) and sulfate (27.22 ± 0.05%) content; therefore, it contains 99.01% fucoidan. According to these results, it is certain that HFPS-F4 can be named as fucoidan.

In order to further investigate the structural characteristics of HFPS-F4, FT-IR and ^1^H NMR spectra of HFPS-F4 were determined. The FT-IR spectra of HFPS-F4 and commercial fucoidan are shown in Figure 2B. The absorption at 845 cm^−1^ indicate a sulfate group at axial C-4 and the shoulder absorption at 820 cm^−1^ indicate a sulfate group at C-2. In addition, the peaks between 1120 and 1270 cm^−1^ indicate the sulfate groups (S=O stretching) branching off from fucoidan backbone [28].

The ^1^H NMR spectra of HFPS-F4 was shown in Figure 2D. The characteristic peaks of sulfated polysaccharide are exhibited in the spectrum. The proton signals from 3.50 to 4.40 ppm were assigned to H2-H6 of the sugar residues [29]. The α-anomeric region (4.9 to 5.4 ppm) was observed and it can be assigned as (1-4)-α-D-Glcp [30]. The signals at the region from 1.2 to 1.5 ppm were assigned to C6 methyl protons of *L*-fucopyranose [29,31]. According to the above results, HFPS-F4 showed the similarities to the fucoidans isolated from brown seaweeds. Therefore, HFPS-F4 was identified as a fucoidan [29,30,31].

Various fucoidans from *H. fusiforme* have been identified in recent years [32,33,34,35,36]. Cheng et al. (2019) has isolated a fucoidan (SFF) from *H. fusiforme* by acid-assisted extraction and investigated the physicochemical property of SFF [33]. The results indicated that SFF contains 68.33% of carbohydrate and 14.55% sulfate contents [33]. SFF has an average Mw of 208.5 kDa, and the carbohydrate of SFF was composed by fucose, rhamnose, glucose, galactose, xylose, mannose, and glucuronic acid with a ratio of 55.67:3.34:5.44:20.83:3.70:4.55 [33]. In addition, further research indicated that SFF improved the liver function and suppressed oxidative stress in streptozotocin-induced diabetic mice [33]. In the present research, we isolated fucoidan form *H. fusiforme* by enzyme-assisted extraction and a fucoidan (HFPS-F4) with a Mw of 102.67 kDa was obtained. Compared with SFF, HFPS-F4 has a lower Mw, and possessed higher carbohydrate (71.79%) and sulfate (27.22%) contents, as well as a higher amount of fucose (79.20%) in the carbohydrates [33]. It indicated that enzyme-assisted extraction could more effectively degradation of carbohydrates in *H. fusiforme* or other plans, compared to acid-assisted extraction. Moreover, these results implied the antioxidant potential of HFPS-F4.

### 3.3. In Vitro Antioxidant Activity of Fucoidan

The in vitro antioxidant activity of the fucoidan obtained from the enzymatic digest of *H. fusiforme* was investigated by evaluating its free radical scavenging activity and effect on H_2_O_2_-induced oxidative stress in Vero cells. As described above, the fucoidan possesses strong free radical scavenging activity. Chen et al. (2016) has investigated the antioxidant activities of crude polysaccharides obtained from *H. fusiforme* (PSF) by boiling water extraction and the H_2_O_2_-degraded polysaccharides (DPSF) obtained from PSF [37]. The results indicated that PSF has an average Mw of 987 kDa; possessed 41.90% of carbohydrate and 14.72% of sulfate; scavenged DPPH radical at the IC_50_ value of 0.61 mg/mL; scavenged 18.33% of hydroxyl radical at the 1.0 mg/mL [37]. Besides, DPSF has an average Mw of 407 kDa; possessed 54.88% of carbohydrate and 16.38% of sulfate; scavenged DPPH radical at the IC_50_ value of 0.27 mg/mL; scavenged 32.98% of hydroxyl radical at the 1.0 mg/mL [37]. These results indicated that the antioxidant activity of the polysaccharides from *H. fusiforme* may relate to its carbohydrate and sulfate composition. In addition, these results were further confirmed for the antioxidant potential of the fucoidan obtained from the enzymatic digest of *H. fusiforme*.

**Figure 3:**
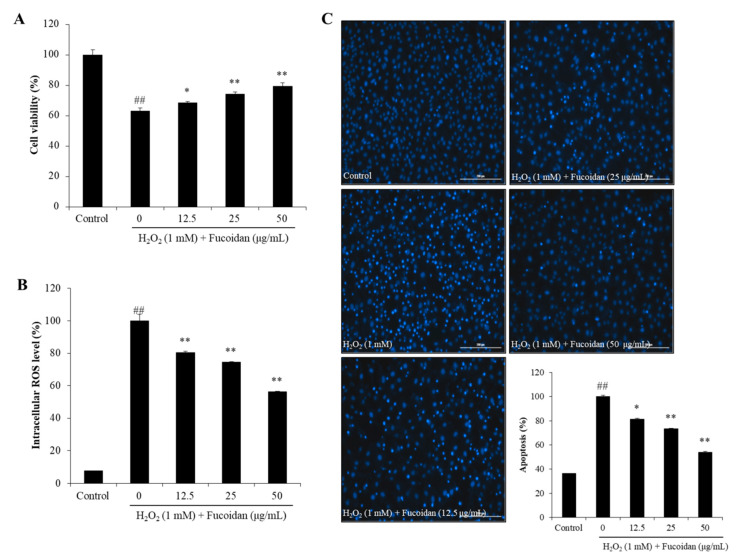
The effect of fucoidan on H_2_O_2_-induced oxidative stress in Vero cells. (**A**) The protective effect of fucoidan against H_2_O_2_-induced cell death in Vero cells; (**B**) the intracellular reactive oxygen species (ROS) scavenging effect of fucoidan in H_2_O_2_-stimulated Vero cells; (**C**) the protective effect of fucoidan against H_2_O_2_-induced apoptosis in Vero cells. The intracellular ROS level was determined by DCF-DA assay and the cell viability was evaluated by MTT assay. The apoptotic body formation was observed under a fluorescence microscope after Hoechst 33342 staining. Apoptosis levels were measured using Image J software. The experiments were conducted in triplicate, and the data are expressed as the mean ± SE. * *p* < 0.05, ** *p* < 0.01 as compared to the H_2_O_2_-treated group and ^##^ *p* < 0.01 as compared to the control group.

In order to investigate the antioxidant effect of fucoidan on H_2_O_2_-induced oxidative stress, we had evaluated the viability, intracellular ROS, and apoptotic body formation of H_2_O_2_-treated Vero cells. As Figure 3A shows, the viability of H_2_O_2_-treated cells (63.00%) was significantly decreased compared to non-H_2_O_2_-treated cells (100%). The viabilities of the cells pretreated with 12.5, 25, and 50 μg/mL of fucoidan were 68.41%, 74.17%, and 79.32%, respectively. This result indicated that the fucoidan dose-dependently increased the viability of H_2_O_2_-treated Vero cells. Previous study displayed that the crude sulfated polysaccharides (HFPS) improved the viabilities of H_2_O_2_-treated Vero cells from 51.22% to 65.53%, 62.80%, and 73.56% at the concentration of 25, 50, and 100 μg/mL respectively [16]. Compared previous and present results we can conclude that the purified fucoidan possesses stronger effect against H_2_O_2_-induced cell death than the crude sulfated polysaccharides.

The intracellular ROS levels of H_2_O_2_-treated Vero cells were shown as Figure 3B. As the results show, H_2_O_2_ significantly stimulated the intracellular ROS generation, whereas, fucoidan remarkably and dose-dependently scavenged ROS. Besides, the apoptotic body formation level of H_2_O_2_-treated Vero cells was determined by Hoechst 33342 stain. As displayed in Figure 3C, the apoptosis level of H_2_O_2_-treated Vero cells was significantly increased, but dose-dependently decreased in fucoidan-treated cells. These results demonstrated that the fucoidan effectively reduces apoptotic body and intracellular ROS levels in H_2_O_2_-treated Vero cells, as well as suggested that the fucoidan prevented cell against H_2_O_2_-induced cell death may through reducing apoptosis via scavenging intracellular ROS.

**Figure 4:**
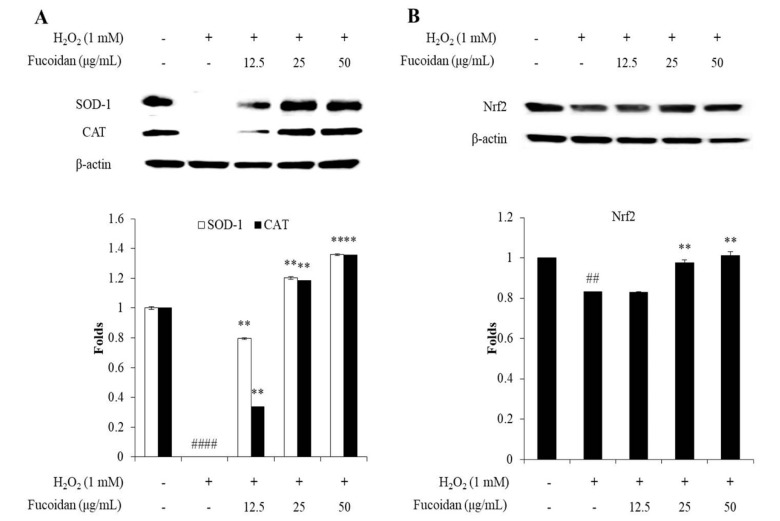
The effect of fucoidan on nuclear factor (erythroid-derived 2)-like 2 (Nrf2) pathway and antioxidant enzymes expression in H_2_O_2_-stimulated Vero cells. (**A**) The expression of catalase (CAT) and superoxide dismutase-1 (SOD-1); (**B**) the expression of Nrf2. The amount of CAT, SOD-1, and Nrf2 were compared with β-actin. “+”: treatment; “-“: non-treatment. The experiments were conducted in triplicate, and the data are expressed as the mean ± SE. * *p* < 0.05, ** *p* < 0.01 as compared to the H_2_O_2_-treated group and ^##^ *p* < 0.01 as compared to the control group.

To investigate the mechanism of fucoidan against H_2_O_2_-induced oxidative stress, the levels of endogenous oxidative stress-related proteins including SOD-1, CAT, and Nrf2 were evaluated. SOD-1 and CAT are endogenous antioxidant enzymes. SOD-1 is the enzyme, which catalyzes the superoxide (O_2_^−^) radical into oxygen (O_2_) and H_2_O_2_. CAT is the enzyme can catalyze H_2_O_2_ to H_2_O and O_2_ [38]. Both enzymes play an important role in oxidative defense. As shown in Figure 4A, H_2_O_2_ significantly reduced CAT and SOD-1 levels. However, fucoidan remarkably and dose-dependently increased the SOD-1 and CAT levels in H_2_O_2_-stimulated Vero cells. This result indicated that the fucoidan scavenged intracellular ROS in H_2_O_2_-stimulated Vero cells may through increasing the level of SOD-1 and CAT.

Nrf2 regulates the expression of antioxidant enzymes such as SOD-1 and CAT. Normally, Nrf2 is located in the cytosol and bound to a control protein that Kelch-like ECH-associated protein 1 (Keap1). In the oxidative stress condition, Nrf2 is released from Keap1, initiates translocation to the nucleus, and active the antioxidant genes to expression antioxidant enzymes [39]. As shown in Figure 4B, the total Nrf2 levels of H_2_O_2_-stimulated Vero cells was significantly decreased, whereas, increased in fucoidan-treated cells in a dose-dependent manner. This result demonstrates that the fucoidan increased the levels of SOD-1 and CAT by up-regulating total Nrf2 level.

### 3.4. In Vivo Antioxidant Activity of Fucoidan

**Figure 5:**
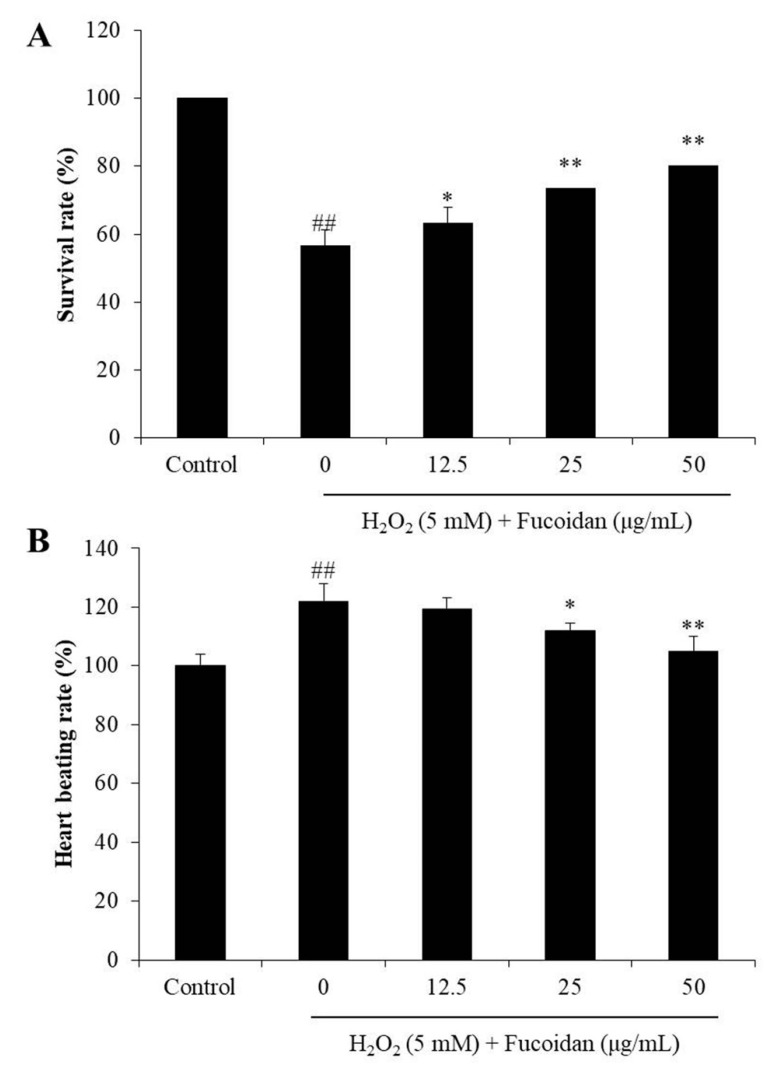
The effect of fucoidan on H_2_O_2_-induced alterations in survival rate and heart-beating rate of zebrafish. (**A**) The survival rate of zebrafish; (**B**) the heart-beating rate of zebrafish. The experiments were conducted in triplicate, and the data are expressed as the mean ± SE. * *p* < 0.05, ** *p* < 0.01 as compared to the H_2_O_2_-treated group and ^##^ *p* < 0.01 as compared to the control group.

Zebrafish is a popular in vivo model in biological, pharmaceutical, and toxicological research. Zebrafish stimulated with H_2_O_2_ has been successfully used to investigate the antioxidant activity of natural products in the previous studies [16]. Therefore, H_2_O_2_-stimulated zebrafish has been selected as an in vivo model to investigate the in vivo antioxidant activity of fucoidan in this study. As shown in Figure 5A, the survival rate of the H_2_O_2_-treated zebrafish (56.66%) was significantly decreased comparing to non-H_2_O_2_-treated zebrafish (100%). However, the survival rates of the zebrafish treated with 12.5, 25, and 50 μg/mL of fucoidan were improved to 63.33%, 73.33%, and 80.00%. In addition, as Figure 5B shows, the heart-beating rate of H_2_O_2_-treated zebrafish was 121%, compared with the control group (100%). Whereas, the heart-beating rate of fucoidan-treated zebrafish was decreased to 119.28%, 112.07%, and 104.97% (Figure 5B). These results indicate that the fucoidan can protect zebrafish against H_2_O_2_-induced damage and heart-beating disorder.

**Figure 6:**
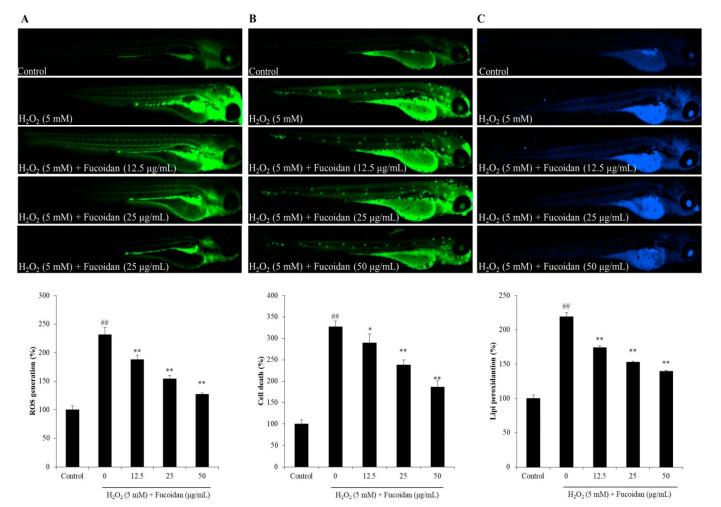
The effect of fucoidan on H_2_O_2_-induced ROS generation, cell death, and lipid peroxidation in zebrafish. (**A**) The protective effect of fucoidan against H_2_O_2_-induced ROS generation; (**B**) the protective effect of fucoidan against H_2_O_2_-induced cell death; (**C**) the protective effect of fucoidan against H_2_O_2_-induced lipid peroxidation. The relative fluorescence intensities of zebrafish were determined using Image J software. The experiments were conducted in triplicate, and the data are expressed as the mean ± SE. * *p* < 0.05, ** *p* < 0.01 as compared to the H_2_O_2_-treated group and ^##^ *p* < 0.01 as compared to the control group.

Furthermore, the ROS production, cell death, and lipid peroxidation of H_2_O_2_-treated zebrafish were measured, and the results were submitted in Figure 6. The results indicated that H_2_O_2_ significantly increased the generation of ROS to 231.33% compared to control group (100%), however, fucoidan reduced ROS level to 187.73%, 154.26%, and 126.89% at the concentration of 12.5, 25, and 50 μg/mL, respectively (Figure 6A). As Figure 6B shows, the cell death of H_2_O_2_-treated zebrafish was 227.31% increased comparing to control group (100%), but, 37.58, 89.46, and 141.26 decreased in the zebrafish treated with 12.5, 25, and 50 μg/mL of fucoidan, respectively. The H_2_O_2_-treated zebrafish revealed 219% of lipid peroxidation, whereas lipid peroxidation of the zebrafish treated with 12.5, 25, and 50 μg/mL of fucoidan showed dramatically decreased to 174.23%, 152.82%, and 139.66%, respectively (Figure 6C). These results indicated that the fucoidan effectively and dose-dependently suppressed H_2_O_2_-induced ROS production, cell death, and lipid peroxidation.

## 4. Conclusions

In the present study, a fucoidan with a Mw of 102.67 kDa was successfully isolated from the sulfated polysaccharide of *H. fusiforme*, which was prepared by Celluclast-assisted extraction and ethanol precipitation. The fucoidan exhibited excellent in vitro antioxidant activities in H_2_O_2_-induced Vero cells. It prevented H_2_O_2_-induced cell death via reducing apoptosis by scavenging intracellular ROS through increasing intracellular SOD-1 and CAT, which are expressed by the up-regulation of Nrf2. In addition, fucoidan demonstrated potent in vivo antioxidant activity indicated in improving the survival rate and decreasing heart-beating rate, as well as reducing ROS, cell death, and lipid peroxidation in H_2_O_2_-stimulated zebrafish. These results suggest that the fucoidan from *H. fusiforme* possess strong in vitro and in vivo antioxidant activities, and may be used as an ingredient in functional food or cosmeceutical industries.

## Figures and Tables

**Table 1:** The chemical composition of HFPS-F4.

Chemical Composition	HFPS-F4
Phenolic content (%)	0.00 ± 0.12
Carbohydrate content (%)	71.79 ± 0.56
Sulfate content (%)	27.22 ± 0.05
Fucoidan * (%)	99.01
Monosaccharide composition (%)	
Fucose	79.20
Rhamnose	2.09
Glucose	0.19
Mannose	18.13
Arabinose	0.38
Molecular weight distribution (kDa)	102.67

* Fucoidan content = carbohydrate content + sulfated content. The experiments were conducted in triplicate, and the data are expressed as the mean ± SE.
